# *Rhodotorula Mucilaginosa*, a Quorum Quenching Yeast Exhibiting Lactonase Activity Isolated from a Tropical Shoreline

**DOI:** 10.3390/s140406463

**Published:** 2014-04-09

**Authors:** Norshazliza Ab Ghani, Joanita Sulaiman, Zahidah Ismail, Xin-Yue Chan, Wai-Fong Yin, Kok-Gan Chan

**Affiliations:** Division of Genetics and Molecular Biology, Institute of Biological Sciences, Faculty of Science, University of Malaya, Kuala Lumpur 50603, Malaysia; E-Mails: norshazliza_shaz@ymail.com (N.A.G.); joanitasulaiman@yahoo.com (J.S.); zahidah_ismail@yahoo.com (Z.I.); xinyuechan@gmail.com (X.-Y.C.); yinwaifong@yahoo.com (W.-F.Y.)

**Keywords:** *N*-hexanoyl-L-homoserine lactone (C6-HSL), *N*-(3-oxo-hexanoyl)-L-homoserine lactone (3-oxo-C6-HSL), *N*-(3-hydroxyhexanoyl)-L-homoserine lactone (3-hydroxy-C6-HSL), quorum sensing, quorum quenching

## Abstract

Two microbial isolates from a Malaysian shoreline were found to be capable of degrading *N*-acylhomoserine lactones. Both Matrix Assisted Laser Desorption Ionization-Time of Flight-Mass Spectrometry and 18S rDNA phylogenetic analyses confirmed that these isolates are *Rhodotorula mucilaginosa*. Quorum quenching activities were detected by a series of bioassays and rapid resolution liquid chromatography analysis. The isolates were able to degrade various quorum sensing molecules namely *N*-hexanoyl-L-homoserine lactone (C6-HSL), *N*-(3-oxo-hexanoyl)-L-homoserine lactone (3-oxo-C6-HSL) and *N*-(3-hydroxyhexanoyl)-L-homoserine lactone (3-hydroxy-C6-HSL). Using a relactonisation assay to verify the quorum quenching mechanism, it is confirmed that *Rh. mucilaginosa* degrades the quorum sensing molecules via lactonase activity. To the best of our knowledge, this is the first documentation of the fact that *Rh. mucilaginosa* has activity against a broad range of AHLs namely C6-HSL, 3-oxo-C6-HSL and 3-hydroxy-C6-HSL.

## 1. Introduction

Quorum sensing (QS) enables microorganisms to communicate via secreted signaling molecules called autoinducers and contributes to the regulation of gene expression in response to bacterial population density [1]. Many Gram-negative bacteria use the *N*-acyl homoserine lactones (AHLs) as autoinducers [2] in which AHLs consist of a 4- to 18-carbon *N*-acyl side chain linked to a lactone ring [3]. AHLs are synthesized by the activity of LuxI synthase using *S*-adenosylmethionine and acylated acyl carrier protein as substrates [4]. The stability of AHLs is pH-dependent, whereby the lactone ring will hydrolyze under alkaline conditions, resulting in a homoserine structure with an opened ring. Such a process is reversible with a switch of pH to acidic conditions [5,6]. Gram-negative bacteria employ AHL as QS signals in their communication circuits to coordinate various physiological activities. These signals lead to an activation of many processes including symbiosis, virulence, competence, conjugation, antibiotic production, motility, sporulation, and biofilm formation [2].

On the other hand, quorum quenching (QQ) is known as a process in which disrupts the QS signals by using several ways which includes enzymatic destruction of the signal molecules, development of antibodies or through QS signalling molecules blocking agents [7–9]. The first documented QQ enzyme was produced by a soil bacterium from the genus *Bacillus* in which was encoded by the *aiiA* gene. It was later characterized as an AHL-lactonase [8]. Since then, these QQ systems have been found in various microorganisms that use them to prevent the benefits of QS in an attempt to gain competitive advantage in polymicrobial environment [10]. The QQ properties of the genera *Bacillus* and *Pseudomonas* have been well established [11]. Examples are the AiiA lactonase homologs of *Bacillus* and PvdQ and also QuiP of *Pseudomonas* [12,13]. These reactions are produced enzymatically by lactonases such as AiiA, AttM, AiiB [14,15] and AhlD [16]. The disruption of QS signaling molecules is considered as a potential way of preventing and treating infections besides preventing plant diseases [17].

In view of this, we have isolated *Rhodotorula mucilaginosa* from a Malaysian shoreline which possesses QQ properties. The genus *Rhodotorula* is known as a saprophytic yeast that can be obtained from environmental sources [18] and has been described as a pathogen antagonist [19]. This study is the first documentation of AHL-degrading activity produced by a marine yeast member from the genus *Rhodotorula*.

## 2. Experimental Section

### 2.1. Sample Collection and Microbial Isolation

A water sample (50 mL) was collected in a sterile container from the shoreline of Batu Maung Penang, Malaysia. The sample was immediately processed upon returning to the laboratory. Water sample was diluted ten-fold and plated onto Luria Bertani (LB) agar. Pure colony was obtained by repeated streaking on LB agar grown at 28 °C.

### 2.2. Isolation and Identification of Microbial Strains

Microbial isolates of interest were identified using a Matrix Assisted Laser Desorption Ionization-Time of Flight-Mass Spectrometer (MALDI-TOF-MS, Bruker, München, Germany) [20] extraction method with a UV laser wavelength of 337 nm and acceleration voltage of 20 kV. Each spot on the target plate was measured by the MBT-autoX.axe autoExecute method. The bacterial spectra were then analyzed in the Bruker MALDI Biotyper Real Time Classification (RTC) Version 3.1 (Build 65) software. In order to verify the isolates identification, phylogenetic analysis was done via 18S rDNA gene nucleotide sequences.

The genomic DNA of the yeast isolates were extracted using the QIAamp^®^ DNA Mini Kit (Qiagen, Frankfurt, Germany) and used as template for PCR. The 18S rDNA gene was PCR-amplified using the following primers namely ITS1 (5′-TCCGTAGGTGAACCTGCGG-3′) and ITS4 (5′-TCCTCCGCTTATTGATATGC-3′) as described previously [21,22]. Nucleotide sequences were compared with GenBank databases using the BLASTN program followed by sequence alignment [23,24]. A phylogenetic tree was generated using the Molecular Evolutionary Genetic Analysis (MEGA) version 5.2 with Neighbour-Joining algorithm and 1,000 re-samplings [25,26].

### 2.3. Rapid Resolution Liquid Chromatography (RRLC) Analysis

Sample preparation for RRLC analysis was performed similar to the whole-cell AHL inactivation assay as described above, with the exception that the final concentration of AHL was 50 μM after rehydration with cell suspensions. AHLs were extracted twice using ethyl acetate, followed by drying by evaporation of the extraction. Acetonitrile (100 μL) was added to the extracted AHL and subjected to RRLC analysis using an Agilent Technologies 1200 series RRLC system [27] (Agilent, Santa Clara, CA, USA). AHL samples were separated in a Agilent Poroshell 120 EC-C18 column (4.6 mm × 100 mm, 2.7 μm particle size) with an elution procedure consisting of an isocratic profile of acetonitrile/water (35:65, v/v) for short chain AHLs with a constant flow rate of 0.7 mL/min and detection wavelength at 210 nm with 0.2. 0.4, 0.6, 0.8 and 1.0 μg/μL of synthetic AHLs were loaded as standards. AHL incubated with *Escherichia coli* TOP10 cells and PBS buffer served as negative controls, while AHL incubated with *Bacillus cereus* acted as positive control. Chromatographs were expressed as milli-absorbance units (mAUs, vertical axis) and time (minutes, horizontal axis), respectively.

### 2.4. Identification of AHL Lactonase Activity

Overnight cultures of QQ yeast cells were harvested by centrifugation. Cell pellets were washed twice and re-suspended in phosphate buffered saline (PBS) (100 mM, pH 6.5). Selected known concentrations of synthetic AHLs (C6-HSL, 3-oxo-C6-HSL and 3-hydroxy-C6-HSL, Sigma-Aldrich, St. Louis, MO, USA) were dispensed into sterile micro-centrifuge tubes and dried by evaporation. Yeast cell suspensions were then added to rehydrate the AHLs to final concentrations of 0.5 μM. The mixtures were then incubated at 28 °C with shaking (220 rpm) for 0 h and 24 h. All reactions were stopped by heat inactivation at 95 °C. For the detection of AHL degradation, 10 μL of reaction mixture was spotted onto sterile paper discs placed on a *Chromobacterium violaceum* CV026 lawn and incubated overnight at 28 °C. AHL inactivation assays involved incubation of *E. coli* TOP10 and PBS buffer as negative controls. Re-lactonisation with acidification using hydrochloric acid (HCl, 0.2 M) was performed as reported [11].

## 3. Results and Discussion

### 3.1. Isolation and Identification of Isolated Strains

**Figure 1.**
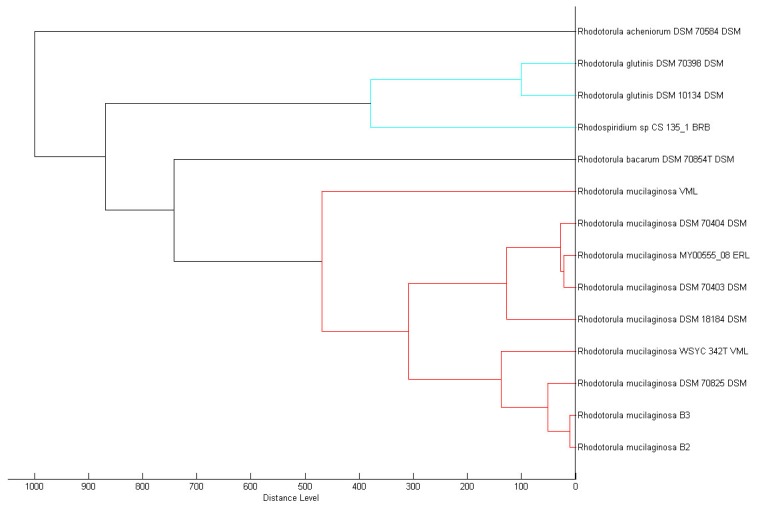
Dendrogram of isolates B2 and B3. Identification of isolates B2 and B3 was analysed using MALDI-TOF-MS and data were presented as dendrogram which indicates these isolates are identified as *Rh. mucilaginosa*.

**Figure 2.**
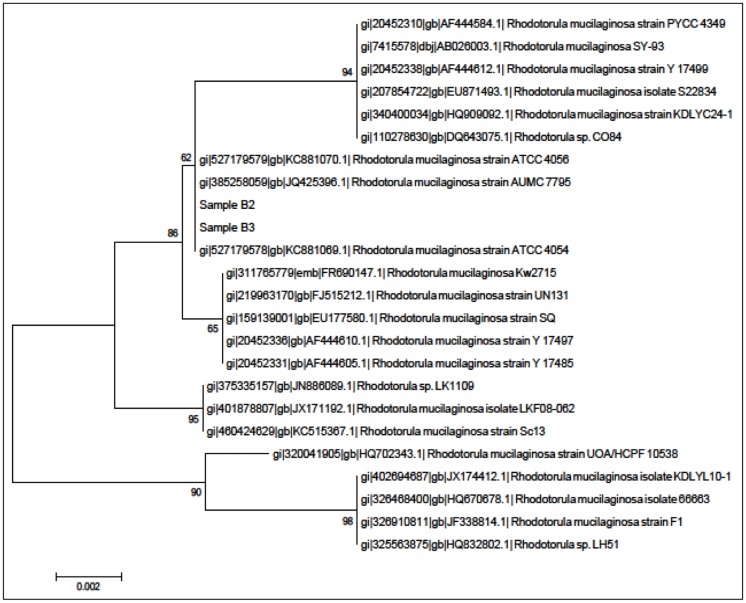
Phylogenetic analysis of isolates B2 and B3 analysed using MEGA 5.2 Molecular identification of isolates B2 and B3 based on 18S rDNA phylogenetic analyses suggested both isolates are closely related to *Rh. mucilaginosa*.

A total of two yeast colonies (isolates B2 and B3) were purified from the tropical Malaysian shoreline. In order to identify the isolates, MALDI-TOF-MS was performed and later verified through 18S rDNA phylogenetic analysis. Both of the MALDI-TOF-MS (Figure 1) and 18S rDNA phylogenetic analyses (Figure 2) identified both isolates belonged to *Rh. mucilaginosa*.

### 3.2. Degradation of AHLs by Rh. Mucilaginosa Isolates B2 and B3

**Figure 3.**
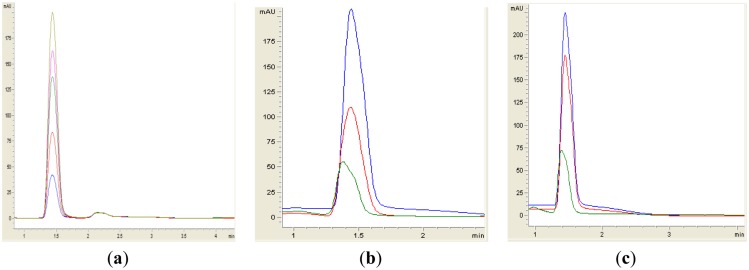

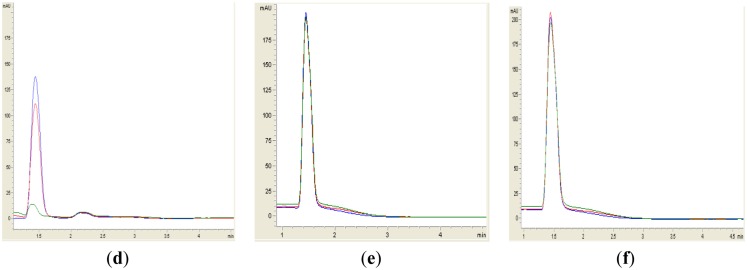
RRLC analysis of C6-HSL degradation. Residual C6-HSL (with elution time of 1.50 min ± 1.2 s), after degradation for 0 h (blue), 24 h (green) and relactonisation with HCL (red) was monitored at 210 nm. Degradation of C6-HSL is depicted by the reduction of milli-absorbance units (mAU) in the chromatogram. (**a**) Synthetic C6-HSL was used at 0.2. 0.4, 0.6, 0.8 and 1.0 μg/μL as standards (corresponding to peaks with ascending height); (**b**) isolate B2; (**c**) isolate B3; (**d**) *B. cereus* as positive control; both (**e**) *E. coli* TOP10; and (**f**) PBS buffer acted as the negative controls. Degradation and relactonisation of C6-HSL was observed by both the B2 and B3 isolates.

**Figure 4.**
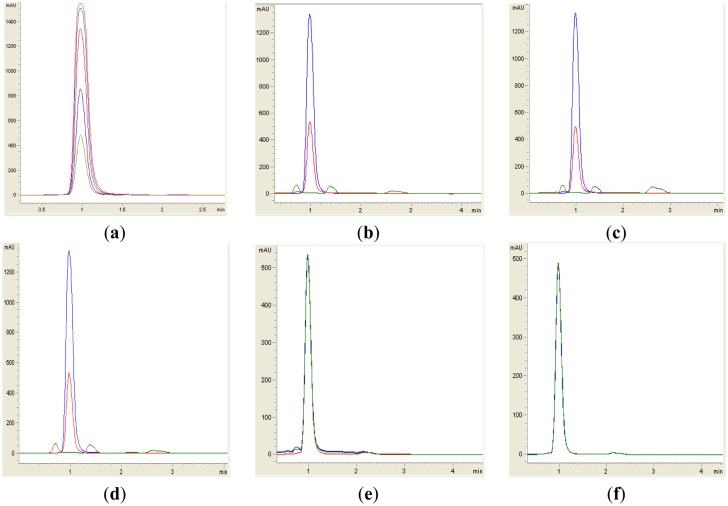
RRLC analysis of 3-oxo-C6-HSL degradation. Residual 3-oxo-C6-HSL (with elution time of 1.00 min ± 1.2 s), after degradation at 0 h (blue), 24 h (green) and relactonisation with HCl (red) was monitored at 210 nm. Degradation of 3-oxo-C6-HSL is depicted by the reduction of milli-absorbance units (mAU) in the chromatogram. (**a**) Synthetic 3-oxo-C6-HSL was used at concentrations of 0.2. 0.4, 0.6, 0.8 and 1.0 μg/μL as standards (corresponding to peaks with ascending height); (**b**) isolate B2, (**c**) isolate B3; (**d**) *B. cereus* as positive control; both (**e**) *E. coli* TOP10; and (**f**) PBS buffer acted as the negative controls. Degradation and relactonisation of 3-oxo-C6-HSL was observed by both the B2 and B3 yeast isolates.

**Figure 5.**
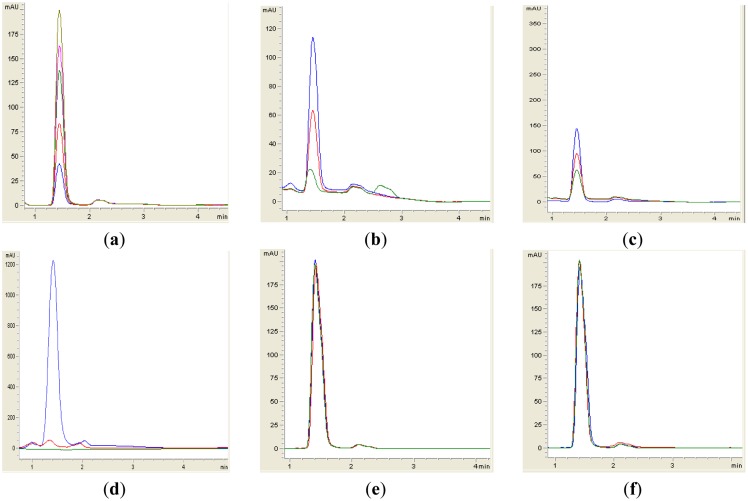
RRLC analysis of 3-hydroxy-C6-HSL degradation. Residual 3-hydroxy-C6-HSL (with elution time of 1.50 min ± 1.2 s), after degradation at 0 h (blue), 24 h (green) and relactonisation with HCl (red), was monitored at 210 nm. Degradation of 3-hydroxy-C6-HSL was depicted by the reduction of milli-absorbance units (mAU) in the chromatogram. (**a**) Synthetic 3-hydroxy-C6-HSL was used at concentrations of 0.2. 0.4, 0.6, 0.8 and 1.0 μg/μL as standards (corresponding to peaks with ascending height); (**b**) isolate B2; (**c**) isolate B3; (**d**) *B. cereus* as positive control; both (**e**) *E. coli* TOP10; and (**f**) PBS buffer acted as the negative controls. Both the B2 and B3 yeast isolates are capable to degrade AHLs via lactonase activity as judged by relactonisation of 3-hydroxy-C6-HSL.

**Figure 6.**
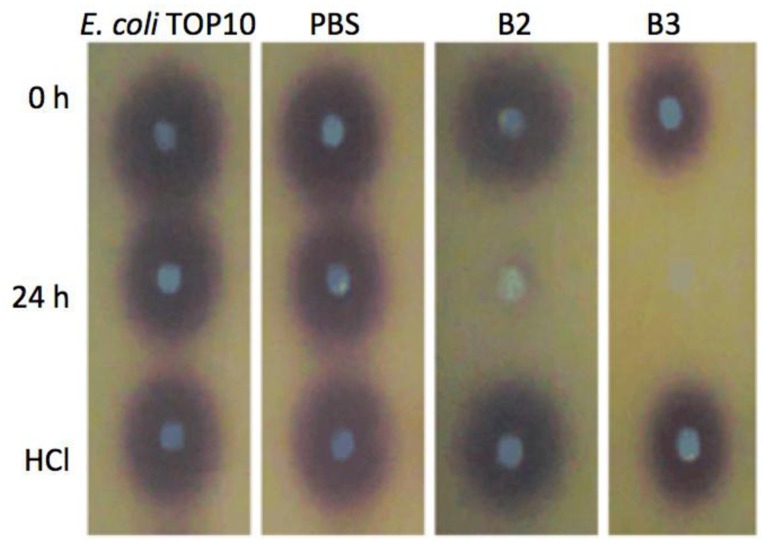
Detection of lactonase activity using CV026 overlay. Yeast suspensions (B2 and B3) were incubated with 3-oxo-C6-HSL for 0 h and 24 h as indicated on the left of figure. Positive QQ activity can be seen as the abolishment of purple pigments after 24 h of incubation. *B. cereus* represents the positive control while *E. coli* TOP10 and PBS buffer acted as negative controls. The purple pigments with the addition of hydrochloric acid (HCl) suggested re-lactonisation of the digested AHL indicated that both the isolates produced lactonase in the presence of 3-oxo-C6-HSL.

Both yeast isolates B2 and B3 showed degradation of C6-HSL (Figure 3), 3-oxo-C6-HSL (Figure 4) and 3-hydroxy-C6-HSL (Figure 5) as depicted by the reduction of the respective peaks in RRLC chromatograms after 24 h of incubation of these AHLs with *Rh. mucilaginosa* cells. To confirm whether both *Rh. mucilaginosa* isolates B2 and B3 degraded AHLs via lactonase activity, we acidified the AHL degradation mixture to promote relactonisation of the opened lactone rings [6]. Formation of purple color pigmentation after the addition of 0.2 M hydrochloric acid indicated lactonase production [28] (Figure 6).

Based on the whole-cell AHL inactivation assays and RRLC analyses on the degradation of various AHL, strong QQ activities were observed among the isolated *Rh. mucilaginosa*. In order to determine whether the *Rh. mucilaginosa* strain B2 and B3 inactivated AHLs through both the cleavage of the acyl chain or via lactonolysis, the B2 and B3 strains were incubated with different AHLs for 24 h. The cells were removed and the supernatant was collected and acidified to pH 2 and incubated for further 24 h. This resulted in the pH-mediated recyclization of any opened lactone ring compounds present [6], which would be subsequently detected using the *C. violaceum* CV026 AHL biosensor [29]. Degradation of C6-HSL, 3-oxo-C6-HSL and 3-hydroxy-C6-HSL was detected in the supernatant after 24 h incubation and recovered by acidification indicated that both B2 and B3 possess lactonase activity.

*Rh. mucilaginosa* has been reported as an emerging pathogen, especially in immunocompromised patients [30,31]. Furthermore, *Rh. mucilaginosa* has also been reported previously to reduce the development of natural decay of apples [32] while it inhibits the growth of *Penicillium expansum* and *Botrytis cinerea* which cause blue and gray mold decay, respectively [33]. We speculate that the QQ activity may play a part in restricting the QS activity of surrounding microorganisms, thus reducing QS-mediated phenotypes responsible for food spoilage. However, further analysis of its QQ mechanism should be conducted to confirm this speculation. Both *Rh. mucilaginosa* and *Pseudomonas* species produce a wide range of antimicrobials which help to confer a high competitive ability and to ensure dominance within their respective microbial communities [34]. It may be that QQ activity also plays a key role in enabling *Rh. mucilaginosa* and other microbial weed species to dominate their respective habitats. Further studies of the biology of QQ are needed to fully characterize the ecophysiological consequences [34]. Recently, QQ yeast *Trichosporon loubieri* has been reported to be isolated from tropical wetland waters and capable to grow on *N*-3-oxo-hexanoyl homoserine as carbon and nitrogen source for growth [35]. This work may indicate eukaryotic cells possesses QQ activity that could compete with the prokaryotic cells that rely on QS for coordinated phenotypes.

To the best of our knowledge, this is the first documentation of *Rh. mucilaginosa* to have exhibited QQ activities. Thus, we believe that the both isolated strains of *Rh. mucilaginosa* have potential as biocontrol agents which would delay food spoilage while novel AHL inactivating enzymes may have utility as therapeutic agents [36,37].

## 4. Conclusions/Outlook

This study confirms the degradation of AHLs by both isolated *Rh. mucilaginosa* strains B2 and B3 via lactonase activity. Our data demonstrated that the isolated yeast strains were able to degrade C6-HSL, 3-oxo-C6-HSL and 3-hydroxy-C6-HSL. Further work includes whole genome sequencing of *Rh. mucilaginosa* in order to identify the QQ gene of these strains.
